# Efficacy and Safety of Underwater Versus Conventional Cold Snare Polypectomy for Small Colorectal Polyps: A Meta-Analysis of Randomized Controlled Trials

**DOI:** 10.14740/gr2146

**Published:** 2026-06-16

**Authors:** Hamza Altal, Mohammad Darweesh, Saba Daher, Usama Abu Heija, Chakradhar Reddy

**Affiliations:** a Department of Internal Medicine, East Tennessee State University Quillen College of Medicine, Johnson City, TN, USA; b Department of Internal Medicine, Division of Gastroenterology, East Tennessee State University Quillen College of Medicine, Johnson City, TN, USA

**Keywords:** Underwater cold snare polypectomy, Cold snare polypectomy, Colorectal polyps, R0 resection, Meta-analysis, Randomized controlled trial

## Abstract

**Background:**

Conventional cold snare polypectomy (CCSP) is the standard technique for removal of small non-pedunculated colorectal polyps. However, incomplete resection remains a concern. Recent data suggested that underwater cold snare polypectomy (UCSP) with no submucosal injection may enhance resection depth and histologic clearance. We aimed to compare the two polypectomy techniques with emphasis on efficacy and safety.

**Methods:**

We systematically searched PubMed, Embase, Cochrane Central, and ClinicalTrials.gov from inception through March 2026, for randomized controlled trials (RCTs) comparing UCSP and CCSP for 4–10 mm non-pedunculated colorectal polyps. The primary outcome was *en bloc* resection. Secondary outcomes included non-R0 resection, intraprocedural bleeding, histologic complete resection (R0), and perforation. Random-effects models using DerSimonian-Laird were used to calculate risk ratios (RRs) with 95% confidence intervals (95% CIs).

**Results:**

Three RCTs met criteria (n = 822 polyps, total; UCSP 418, CCSP 404). *En bloc* resection was higher in UCSP compared to CCSP (RR 1.03, 95% CI 1.00–1.06, I^2^ = 0%). UCSP increased histologic complete resection (RR 1.15, 95% CI 1.01–1.31, I^2^ = 81.8%). Adverse events were uncommon; intraprocedural bleeding was similar between groups (RR 0.57, 95% CI 0.16–2.07, I^2^ = 29.5%). No perforations were reported in any arm.

**Conclusions:**

UCSP improves *en bloc* resection of small polyps without increasing adverse events. Further multicenter trials with longitudinal follow-up are needed to assess long-term residual disease.

## Introduction

Colon cancer remains a leading cause of cancer-related mortality worldwide, and the adenoma-carcinoma sequence highlights the importance of complete polyp removal during colonoscopy [1, 2]. Most polyps encountered at screening colonoscopy are small (≤ 10 mm) and carry extremely low malignancy rates; however, incomplete resection of these lesions remains common, with a reported incomplete resection rate of approximately 10% in the complete adenoma resection (CARE) study, and is recognized as an important contributor to interval (post colonoscopy) colorectal cancer risk [3]. Cold snare polypectomy (CSP) has become the guideline recommended standard technique for small polyp removal, given its satisfactory safety profile and evasion of electrocautery-associated thermal injury [4, 5]. The European Society of Gastrointestinal Endoscopy (ESGE) recommends CSP for sessile polyps measuring 5–9 mm [6].

Despite these recommendations, *en bloc* resection rates with conventional CSP (CCSP) can be suboptimal, and histologically verified complete (R0) resection rates vary widely from 43% to 96% across published studies [7–9]. Piecemeal retrieval, specimen breakup during suction, and struggle capturing adequate circumferential margins in an air-distended colon all have contributed to incomplete resection [10]. *En bloc* resection is a critical quality metric because it conserves specimen integrity for precise pathological margin examination and is associated with lower rates of residual adenoma at surveillance [11].

Underwater endoscopic techniques, first described by Binmoeller and colleagues for large colorectal lesions [12], leverage numerous benefits of water immersion. In a water-filled lumen, the colon wall becomes non-distended, the mucosa and submucosa separate from the muscularis propria, and lesions appear more protuberant which facilitates snare capture of these lesions [13, 14]. The water’s higher refractive index compared to air offers visual magnification that enhances identification of polyp margins [15]. These properties have been used in underwater endoscopic mucosal resection (UEMR) with established benefits for larger polyps [16, 17].

Underwater cold snare polypectomy (UCSP) expands these principles to small polyps without electrocautery. Theoretically, the buoyancy effect and enhanced margin identification allow the snare to capture a wider circumferential margin of normal mucosa, thus increasing *en bloc* and complete histologic resection rates. Several recent RCTs have compared UCSP to CCSP [18–20], but no meta-analysis has synthesized this randomized evidence.

We aimed to pool data from RCTs comparing UCSP and CCSP for small (4–10 mm) non-pedunculated colorectal polyps in terms of efficacy and safety.

## Materials and Methods

### Protocol and registration

Our meta-analysis was conducted and reported in accordance with the Preferred Reporting Items for Systematic Reviews and Meta-Analyses (PRISMA) 2020 guidelines [21]. The protocol was not registered prospectively in PROSPERO.

### Eligibility criteria

Inclusion criteria were: RCT design, enrolled adult patients (≥ 18 years) undergoing colonoscopy with non-pedunculated colorectal polyps measuring 4–10 mm, compared UCSP with CCSP, and reported at least the primary outcome of *en bloc* resection. Observational studies, propensity-score-matched analyses, studies limited to polyps > 10 mm, and studies using electrocautery in either arm were excluded.

### Information sources and search strategy

A systematic literature search was performed across PubMed/MEDLINE, Embase, the Cochrane Central Register of Controlled Trials (CENTRAL), and ClinicalTrials.gov from database inception through March 2026. The search strategy combined Medical Subject Headings (MeSH) and free-text terms including: (“underwater” OR “water immersion”) AND (“cold snare” OR “cold snare polypectomy”) AND (“colorectal” OR “colon” OR “polyp”). No language restrictions were applied. Reference lists of included studies and relevant reviews were hand-searched to identify additional eligible trials.

### Study selection and data extraction

Two reviewers independently screened titles, abstracts, and full-text articles for eligibility. Disagreements were set by discussion with a third reviewer. Data were extracted into a standardized form recording: study characteristics (first author, year, country, registration, design, blinding), population characteristics (number of patients and polyps, polyp size range, morphology, histology), intervention details (snare type, snare diameter, technique protocol), and outcomes (*en bloc* resection, R0 resection adverse events, muscularis mucosa and submucosa resection rates, procedure times). The unit of analysis was the polyp for all outcomes.

### Risk of bias assessment

The risk of bias in included trials was assessed using the Cochrane Risk of Bias 2.0 (RoB 2) tool across five domains: randomization process, deviations from intended interventions, missing outcome data, measurement of the outcome, and selection of the reported result [22]. Two reviewers independently measured each domain as low risk, some concerns, or high risk, with disputes solved by discussion with a third author.

### Statistical analysis

Pooled risk ratios (RRs) with 95% confidence internals (CIs) were calculated using DerSimonian-Laird random-effects models [23]. Statistical heterogeneity was quantified using the I^2^ statistic, with values of < 25%, 25–50%, and > 50% representing low, moderate, and substantial heterogeneity, respectively [24]. Due to the small number of included studies, formal assessment of publication bias via funnel plots or Egger’s test was not performed [25]. All analyses were performed in R (version 4.3.2). P value < 0.05 was deemed statistically significant.

### Outcomes

The primary outcome was *en bloc* resection, defined as single-piece removal of the polyp without fragmentation. Key secondary outcomes included: histologically verified complete resection (R0: negative margins per the individual trial’s definition), and adverse events (specifically intraprocedural bleeding, delayed bleeding, and perforation). Polyp histology was extracted as reported by each trial according to the World Health Organization classification of tumors of the digestive system. Polyps were categorized as conventional adenomas (tubular, tubulovillous, or villous), sessile serrated lesions, or hyperplastic polyps, with epithelial dysplasia graded as low-grade or high-grade. Because *en bloc*, single piece resection is a prerequisite for valid histologic margin assessment, R0 (complete) resection was evaluated only in specimens amenable to margin evaluation; fragmented or piecemeal specimens with indeterminate margins were categorized as non-R0 (incomplete) resection.

### Ethical compliance

This study is a meta-analysis of previously published RCTs and did not involve direct human or animal subjects research. Each included trial obtained its own institutional review board approval and informed consent as described in their respective publications.

## Results

### Study selection

The search yielded 127 records. After removal of duplicates, title/abstract screening, and full-text review, three RCTs met all inclusion criteria [18–20]. A comprehensive search of ClinicalTrials.gov, ICTRP, and the reference lists of recent network meta-analyses [26] identified no additional completed or ongoing RCTs comparing UCSP and CCSP for small polyps. The three included trials enrolled a total of 502 patients with 822 polyps (418 UCSP, 404 CCSP).

### Study characteristics

The characteristics of the included studies are summarized in Table 1. All three trials were prospective, single-center RCTs conducted in South Korea (Myung et al 2022), Greece (Zachou et al 2024, the COLDWATER study), and China (Fu et al 2025). Polyp sizes ranged from 4 to 10 mm across studies. Randomization was performed at the patient level with all eligible polyps in each patient assigned to the same technique. All three trials used dedicated cold snares (9–10 mm diameter), and pathologists were blinded to the resection technique in all studies.

**Table 1:** Studies Characteristics

Study	Country	Design	Patients	Polyps (UCSP/CCSP)	Size (mm)	Snare (mm)
Myung et al, 2022	South Korea	Single-center RCT	110	98/100	4–9	9
Zachou et al, 2024	Greece	Single-center RCT	220	198/199	5–10	10
Fu et al, 2025	China	Single-center RCT	172	122/105	4–9	Not reported

CCSP: conventional cold snare polypectomy; RCT: randomized controlled trial; UCSP: underwater cold snare polypectomy.

Definitions of key outcomes varied across trials. Myung et al defined R0 as *en bloc* resection with histologically clear margins on the specimen. Zachou et al used the Residual Tumor Classification system, supplemented by margin biopsies for Rx specimens. Fu et al defined R0 as negative biopsies from the wound base and four-quadrant margins after polypectomy. *En bloc* resection was consistently defined as single-piece removal across all studies.

The histopathological distribution of resected lesions was balanced between the UCSP and CCSP arms in all three trials, and conventional adenomas were the predominant lesion type across the three studies (Table 2). In Myung et al, most lesions were tubular adenomas with low-grade dysplasia (UCSP 76.5%, CCSP 75.0%); hyperplastic polyps accounted for approximately 20% of each arm, and high-grade dysplasia was isolated (UCSP 1.0%, CCSP 0%). Fu et al enrolled almost exclusively low-grade dysplastic adenomas (UCSP 96.7%, CSP 95.2%) after excluding hyperplastic and sessile serrated lesions during the trial, with high-grade dysplasia in 1.6% and 4.8%, respectively. In Zachou et al (COLDWATER), tubular adenomas predominated (UCSP 51.0%, CCSP 54.3%), followed by tubulovillous adenomas (31.8% vs. 24.1%) and sessile serrated lesions (14.7% vs. 17.6%). No between-arm difference in histology reached statistical significance (Fu et al, P = 0.172; Zachou et al, P = 0.31).

**Table 2:** Histopathological Categorization of Resected Polyps by Study and Treatment Arm

Histological category	UCSP, n (%)	CCSP, n (%)
Myung et al, 2022 (UCSP, n = 98; CCSP, n = 100)		
Tubular adenoma, low-grade dysplasia	75 (76.5)	75 (75.0)
Tubular adenoma, high-grade dysplasia	1 (1.0)	0 (0.0)
Sessile serrated lesion	2 (2.0)	5 (5.0)
Hyperplastic polyp	20 (20.4)	20 (20.0)
Fu et al, 2025 (UCSP, n = 122; CSP, n = 105; P = 0.172)		
Adenoma, low-grade dysplasia	118 (96.7)	100 (95.2)
Tubulovillous adenoma	2 (1.6)	0 (0.0)
Adenoma, high-grade dysplasia	2 (1.6)	5 (4.8)
Zachou et al, 2024/COLDWATER (UCSP, n = 198; CCSP, n = 199; P = 0.31)		
Tubular adenoma	101 (51.0)	108 (54.3)
Villous adenoma	5 (2.5)	8 (4.0)
Tubulovillous adenoma	63 (31.8)	48 (24.1)
Sessile serrated lesion	29 (14.7)	35 (17.6)

CCSP: conventional cold snare polypectomy; UCSP: underwater cold snare polypectomy.

### Risk of bias

Risk of bias assessments are presented in Table 3. All three trials had low risk of bias for the randomization process. Blinding of endoscopists was not possible due to the nature of the intervention. However, pathological assessment was blinded in all studies. Zachou et al was graded low risk across all domains given its prospective trial registration, published protocol, and single-blind design. Myung et al and Fu et al were deemed as having some concerns in the domain of deviations from intended interventions due to the open-label design for operators.

**Table 3:** Risk of Bias Assessment

Study	Randomization	Deviations	Missing data	Outcome measurement	Selective reporting
Myung et al, 2022	Low	Some concerns	Low	Low	Low
Zachou et al, 2024	Low	Low	Low	Low	Low
Fu et al, 2025	Low	Some concerns	Low	Low	Low

### Primary outcome: *en bloc* resection

**Figure 1:**
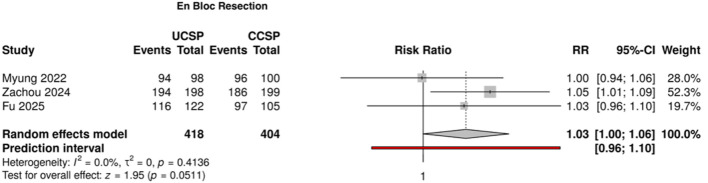
Forest plot for *en bloc* resection.

UCSP was associated with a significantly higher *en bloc* resection rate: (RR 1.03, 95% CI 1.00–1.06, I^2^ = 0%) (Fig. 1). There was no heterogeneity, indicating a consistent effect across all three trials.

### Secondary outcomes

**Figure 2:**
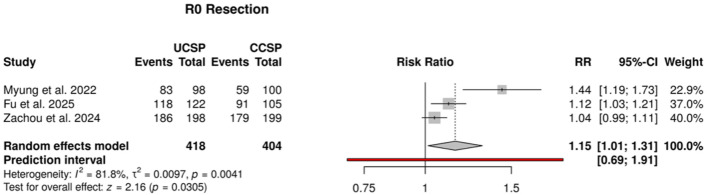
Forest plot for R0 resection.

UCSP was associated with significantly higher R0 (complete histologic) resection (RR 1.15, (95% CI 1.01–1.31, P = 0.031, I^2^ = 81.8%) (Fig. 2). Substantial heterogeneity was observed, driven primarily by the markedly lower CCSP R0 rate in Myung et al (59.0%) compared with the other two studies (≥ 87%). In sensitivity analysis excluding Myung et al, heterogeneity was 0% and the RR remained significant (1.06, 95% CI 1.01–1.12, I^2^ = 0.0%).

**Figure 3:**
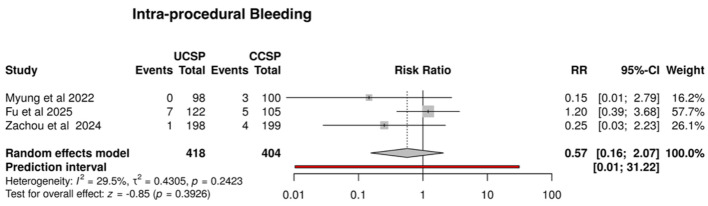
Forest plot for intraprocedural bleeding.

Intraprocedural bleeding was similar between UCSP and CCSP (pooled RR 0.57, 95% CI 0.16–2.07; P = 0.3926). Heterogeneity was low (I^2^ = 29.5%) (Fig. 3).

No delayed bleeding events occurred in UCSP arms across all three studies (vs. one event in Myung et al’s CCSP arm). No perforations occurred in any study arm, and no patients required hospitalization for adverse events.

Myung et al reported significantly shorter total procedure time with UCSP (103.9 ± 35.0 vs. 130.7 ± 80.0 s; P = 0.031), attributed to easier specimen retrieval underwater. Fu et al found no significant difference (109.5 vs. 110.0 s; P = 0.890). Zachou et al did not record procedure times.

## Discussion

This is the first meta-analysis of RCTs comprising 822 polyps that compares UCSP and CCSP for small non-pedunculated colorectal polyps. The principal finding is that UCSP significantly improves *en bloc* resection (RR 1.03, 95% CI 1.00–1.06, I^2^ = 0%), with a consistent effect across all three trials and no statistical heterogeneity. UCSP also improved histologically verified complete (R0) resection (RR 1.15, 95% CI 1.01–1.31), without increasing adverse events. These findings suggest UCSP as a promising enhancement of the CSP technique for small polyps.

*En bloc* resection is a clinically important outcome because single-piece retrieval preserves specimen architecture, enabling accurate histopathological diagnosis and margin assessment while reducing the risk of residual adenoma [11]. Piecemeal removal, by contrast, introduces tissue fragmentation that can lead to margin indetermination and falsely increase incomplete histologic resection rates even when the polypectomy was complete. The improvement in *en bloc* rates with UCSP is mechanically attributable to the physical properties of water immersion as in a non-distended water-filled colonic lumen, the polyps become more protuberant, and the snare can capture the entire lesion with surrounding normal mucosa in a single closure and reduces the likelihood of tissue shearing or partial capture [12–14, 27–29]. Essentially, *en bloc* resection is a prerequisite for a acceptable R0 determination: in a fragmented or piecemeal specimen the lateral and deep margins cannot be reliably examined, so histologic completeness becomes compromised and such specimens are necessarily classified as non-R0. The two efficacy endpoints are therefore mechanistically linked rather than independent; by increasing the proportion of intact specimens, single-piece specimens, UCSP also increases the proportion of lesions in which R0 status can be confidently assessed. This interdependence should be kept in mind when interpreting the pooled R0 estimate, particularly for trials such as Myung et al in which specimen fragmentation occurred exclusively in the CCSP arm (5.3% vs. 0%).

The parallel improvement in R0 resection, though complicated by substantial heterogeneity (I^2^ = 81.8%), further supports the efficacy advantage of UCSP. The heterogeneity was primarily driven by the Myung et al, in which the CCSP R0 rate was only 59.0% which is significantly lower than in Zachou et al (90.0%) and Fu et al (86.7%). Several factors may explain this difference. The first, R0 definitions varied: Myung et al relied exclusively on specimen margin assessment, whereas the other two trials complemented this with post-polypectomy margin biopsies. Second, Myung et al reported a 5.3% specimen fragmentation rate with CCSP vs. 0% with UCSP, which directly compromises the ability to histologically examine the margin. Third, the smaller mean polyp size in Myung et al (5.6–5.9 mm) may have increased the difficulty of achieving clear margins on smaller specimens. When this trial was excluded in sensitivity analysis, the heterogeneity was 0% and the R0 advantage persisted (RR 1.06, 95% CI 1.01–1.12), confirming the robustness of the effect.

Beyond resection quality, the procedural characteristics of UCSP appear favorable. Myung et al demonstrated significantly shorter total procedure time with UCSP (103.9 ± 35.0 vs. 130.7 ± 80.0 s; P = 0.031), attributed mainly to easier specimen retrieval in a water-filled lumen where resected tissue floats and can be suctioned easily. Fu et al found no significant difference in procedure time, suggesting that the additional step of water infusion does not meaningfully prolong the procedure. The lesion retrieval advantage is clinically relevant as failed polyp retrieval precludes histopathological evaluation which can necessitate shortened surveillance intervals and increases patient burden. In the Myung et al trial, the retrieval rate was 100% with UCSP versus 94.5% with CCSP (P = 0.030), and specimen fragmentation happened solely in the CCSP arm (5.3% vs. 0%; P = 0.027). These real advantages may support adoption of UCSP in routine clinical practice.

The safety profile was assuring. Intraprocedural bleeding rates were low and similar (RR 0.57, 95% CI 0.16–2.07), and no perforations occurred.

A noteworthy finding from the COLDWATER trial (Zachou et al) was that UCSP produced consistent R0 resection rates irrespective of endoscopist experience, whereas CCSP performance was significantly operator-dependent (expert R0: 93% vs. non-expert: 84%, P = 0.04). UCSP eliminated this gap (expert 94.2% vs. non-expert 93.2%; P = 0.7), suggesting that water immersion balances for operator-dependent variability in snare placement. This finding has possible implications for training programs and could make UCSP a preferred technique for less experienced endoscopists.

### Limitations

Several limitations should be acknowledged. First, only three single-center RCTs were available, limiting generalizability. Second, outcome definitions (particularly R0) varied across the studies, contributing to clinical heterogeneity in the R0 analysis, though the primary outcome of *en bloc* resection was consistently defined and showed no heterogeneity. Third, long-term recurrence data were limited; only Zachou et al reported 12-month follow-up, with significant follow-up loss due to the COVID-19 pandemic. Fourth, the small number of studies prevented formal assessment of publication bias. Fifth, all polyps in each patient were allocated to the same technique, commencing a potential within patient clustering that was not accounted for statistically in the individual trials.

### Conclusions

UCSP significantly improves *en bloc* resection of small non-pedunculated colorectal polyps compared with CCSP with comparable safety. Larger multicenter RCTs with standardized pathological assessment of complete resection, long-term recurrence endpoints, and cost-effectiveness analyses are needed to establish the definitive role of UCSP in our clinical practice.

## Data Availability

All data generated or analyzed during this study are included in this published article. The datasets used are derived from publicly available published randomized controlled trials [18-20].

## Abbreviations

CCSP: conventional cold snare polypectomy
CENTRAL: Cochrane Central Register of Controlled Trials
CI: confidence interval
COLDWATER: a named randomized controlled trial (Zachou et al, 2024)
CSP: cold snare polypectomy
ESGE: European Society of Gastrointestinal Endoscopy
ICTRP: International Clinical Trials Registry Platform
MeSH: Medical Subject Headings
PRISMA: Preferred Reporting Items for Systematic Reviews and Meta-Analyses
PROSPERO: International Prospective Register of Systematic Reviews
R0: histologically verified complete resection with negative margins
RCT: randomized controlled trial
RoB 2: Cochrane Risk of Bias 2.0 tool
RR: risk ratio
UCSP: underwater cold snare polypectomy
UEMR: underwater endoscopic mucosal resection
